# Gene-based SNP discovery in tepary bean (*Phaseolus acutifolius*) and common bean (*P. vulgaris*) for diversity analysis and comparative mapping

**DOI:** 10.1186/s12864-016-2499-3

**Published:** 2016-03-15

**Authors:** Neha Gujaria-Verma, Larissa Ramsay, Andrew G. Sharpe, Lacey-Anne Sanderson, Daniel G. Debouck, Bunyamin Tar’an, Kirstin E. Bett

**Affiliations:** Department of Plant Sciences, University of Saskatchewan, 51 Campus Dr., Saskatoon, SK S7N 5A8 Canada; National Research Council Canada, 110 Gymnasium Place, Saskatoon, SK S7N 0W9 Canada; Genetic Resources Program, International Center for Tropical Agriculture, Km 17 recta a Palmira, AA6713 Cali, Colombia

## Abstract

**Background:**

Common bean (*Phaseolus vulgaris*) is an important grain legume and there has been a recent resurgence in interest in its relative, tepary bean (*P. acutifolius*), owing to this species’ ability to better withstand abiotic stresses. Genomic resources are scarce for this minor crop species and a better knowledge of the genome-level relationship between these two species would facilitate improvement in both. High-throughput genotyping has facilitated large-scale single nucleotide polymorphism (SNP) identification leading to the development of molecular markers with associated sequence information that can be used to place them in the context of a full genome assembly.

**Results:**

Transcript-based SNPs were identified from six common bean and two tepary bean accessions and a subset were used to generate a 768-SNP Illumina GoldenGate assay for each species. The tepary bean assay was used to assess diversity in wild and cultivated tepary bean and to generate the first gene-based map of the tepary bean genome. Genotypic analyses of the diversity panel showed a clear separation between domesticated and cultivated tepary beans, two distinct groups within the domesticated types, and *P. parvifolius* was confirmed to be distinct. The genetic map of tepary bean was compared to the common bean genome assembly to demonstrate high levels of collinearity between the two species with differences limited to a few intra-chromosomal rearrangements.

**Conclusions:**

The development of the first set of genomic resources specifically for tepary bean has allowed for greater insight into the structure of this species and its relationship to its agriculturally more prominent relative, common bean. These resources will be helpful in the development of efficient breeding strategies for both species and will facilitate the introgression of agriculturally important traits from one crop into the other.

**Electronic supplementary material:**

The online version of this article (doi:10.1186/s12864-016-2499-3) contains supplementary material, which is available to authorized users.

## Background

Common bean (*Phaseolus vulgaris* L.) is the most widely grown and consumed grain legume in the world. Despite being a tropical-season legume, it has been adapted to a wide range of environments from Canada to South America, northern Europe to Southern Africa [1]. Limits to adaptation are primarily due to photoperiod sensitivity and sensitivity to various abiotic stresses, particularly to drought and extremes in temperature, both hot and cold.

Tepary bean (*Phaseolus acutifolius* Gray) was also domesticated as a crop and has been produced primarily in the arid regions of southwestern USA and northwestern Mexico for centuries [2, 3]. Being much more heat, drought and salinity tolerant than their common bean relatives, tepary bean cultivars have also been grown in marginal areas of South America and Africa where common bean cannot be grown [4–6]. There has been a resurgence in interest in this crop recently as it is viewed as a potential source of genes for stress tolerance for common bean breeding, with a view to making common bean more resilient in the face of climate instability. It also has the potential as a crop in its own right but currently suffers from a lack of breeding activity directed towards larger seed size and increased yield.

Tepary bean is considered a member of the tertiary gene pool of common bean, and the first few generations following hybridization with *P. vulgaris* generally require embryo rescue to be successful. Tepary bean is the source of tolerance to common bacterial blight found in many cultivars grown today [7–9]. Phylogenetically, both species fall into the Vulgaris group of *Phaseolus* clade B, along with *P. lunatus, P. coccineus* and *P. dumosus* [10]. There are four forms of *P. acutifolius*: cultivated and wild var. *acutifolius*, weedy var. *latifolius* and wild var. *tenuifolius* [11]. *P. parvifolius* is a sister species to *P. acutifolius* and is often confused with var. *tenuifolius* as they are morphologically similar [12]. Cytogenetic analysis suggests strong shared synteny between *P. acutifolius* and *P. vulgaris* and predicts only a few major inversions differentiate the two [13, 14].

Marker-assisted selection (MAS) has been used in common bean breeding programs since the 1990’s. Early markers were based on RAPDs or SCARs derived from these polymorphic fragments. SCAR markers are still routinely used in breeding programs, particularly the SU91 marker for common bacterial blight (CBB) tolerance derived from tepary bean [15]. A large number of SSR markers have been developed for common bean and used extensively for mapping and marker discovery [16–19]. These older forms of markers are not ideal for high throughput marker screening as they are expensive and often cross-specific. They are also limited in their use for comparative mapping, as they do not transfer across species readily.

With the advent of next-generation sequencing (NGS), single nucleotide polymorphisms (SNPs) have become more practical for genotyping and marker discovery. MAS using SNP technology is much quicker and less expensive than older, gel-based systems and is rapidly becoming the marker system of choice in breeding programs. Common bean SNP markers have been developed and used to map populations derived from crosses between Andean and Mesoamerican parents [20, 21] or look at diversity [22–27]. Most of these SNPs were identified by comparing sequence fragments from genotypes belonging to the Andean and Mesoamerican gene pools. This results in low levels of polymorphism within gene pools making their utility limited in breeding programs where most crosses are restricted to one or other of the pools [25].

The recent completion of the common bean genome sequence [28] has facilitated SNP discovery and mapping by allowing short reads from different genotypes to be assembled using the genome sequence as a template. This is more reliable than *de novo* assembly and SNPs can automatically be identified relative to the reference sequence. Furthermore, homologous sequences from closely related species can be aligned to the genome sequence and SNPs that are unique to each species can be identified. These SNPs are much more likely to be useful when studying related species and there is no ascertainment bias when interpreting the data.

Fewer resources have been devoted to tepary bean genomics than to common bean. As of early 2015, only 54,917 tepary bean EST sequences were publically available. Two AFLP combinations [29] and 20 SSR markers [26] have been screened in this species to assess genetic diversity in the germplasm collection at the International Centre for Tropical Agriculture (CIAT). Goretti et al. [22] included four tepary bean genotypes in a panel assessing SNP diversity in *Phaseolus* spp. To date, no SNP resources or any genetic linkage maps have been developed specifically for this crop. Owing to the increased interest in the agriculture value of tepary beans, this study was dedicated to the development of genomic resources for genetics and breeding programs. These genomic tools will also be useful for genetic enhancement of common bean through the tracking of introgressions of desirable alleles from this important relative.

Here we describe the identification of SNPs in both tepary and common bean, the development of two 768-SNP Illumina GoldenGate arrays: one for tepary bean and one for common bean, and the use of the tepary bean array to develop a map of the genome allowing for comparative mapping with common bean.

## Methods

### Plant material

Six common bean and two tepary bean genotypes were selected for sequencing (Table 1). A single plant of each genotype was selfed to produce sufficient seed to plant to generate tissues for library construction. Common bean genotypes included: (i) CDC WM-2: an early maturing, CBB tolerant, indeterminate, slow-darkening pinto bean cultivar released in 2009 from the Crop Development Centre (CDC), University of Saskatchewan [30], (ii) Expresso: an early maturing, determinate black bean cultivar released in 1994 from the CDC (P. Hucl, pers. comm.), (iii) Higuera-E: an early maturing, off-type found in a sample of yellow seeded Higuera-type beans from Mexico (A. Vandenberg, pers. comm.), (iv) SMARC1N-PN1: a navy bean mutant with an altered protein profile [31], (v) BAT 93 and (vi) Jalo EEP-558: the Mesoamerican and Andean parents, respectively, of the original core mapping population for common bean [32]. The tepary bean genotypes were (i) W6 15578, and (ii) PI 430219 identified as contrasting in tolerance to sub-zero temperatures [33] and parents of an F_2_ mapping population.

**Table 1:** Common and tepary bean genotypes and SNP statistics

Genotype	Market class	Race	Total reads	Total uniquely mapped reads	# of Pv0.9 scaffolds mapped to	Total SNPs	Average read depth
CDC WM-2	Pinto	Mesoamerican	1007453	783163	2287	52640	18.6
Expresso	Black	Mesoamerican	613558	389281	1687	43308	10.2
Higuera-E	Yellow	Andean	568091	477595	1755	9516	13.8
SMARC1N-PN1	Navy	Mesoamerican	517863	442403	1760	45076	9.9
Jalo-EEP-558	Red	Andean	623709	475570	2227	9550	11.9
BAT 93	Black	Mesoamerican	509572	323613	1889	37938	9.4
W6 15578		*P. acutifolius* spp. *acutifolius*	556903	355707	1939	164162	12.6
PI 430219		*P. acutifolius* spp. *tenuifolius*	590067	438042	1928	171524	16.4

An F_2_ mapping population (BR-06) of 186 individuals was developed from a cross between the two tepary bean accessions, W6 15578 and PI 430219, for use in genetic mapping. A diverse set of 94 domesticated tepary bean genotypes was obtained from the Genetic Resources Program at CIAT and DNA for 96 domesticated and wild tepary beans was obtained from the USDA-ARS Mayaguez, PR. Four tepary bean cultivars were obtained from Prairie Garden Seeds in Saskatchewan (SK), Canada. There were 49 accessions in common between the CIAT and USDA-ARS sets, resulting in a set of 158 unique lines (Additional file 1).

All plants were grown in a controlled environment chamber at the University of Saskatchewan. DNA was extracted from freeze-dried leaf tissues of BR-06 and its parents, as well as a pool of leaf tissue from five plants of the accessions from CIAT and SK, using a modified CTAB method [34]. The quality and quantity of DNA were assessed using a FLUOstar Omega fluorimeter (BMG Labtech). DNA was normalized to 50 ng/μL for SNP genotyping.

### RNA isolation, cDNA construction and sequencing

Several plants of each of the six common bean and two tepary bean genotypes were grown in controlled environment chambers and tissue was harvested from each at various stages, including 2-week old leaf, stem before flowering, 1-week old etiolated seedling, mixed flower stages, and developing seed at mixed stages. RNA extractions, cDNA synthesis, 3’-anchored cDNA library construction and Roche 454 Titanium sequencing were performed as described in Sharpe et al. [35]. These libraries were sequenced at the National Research Council Canada, Saskatoon, SK, Canada using the high throughput Roche 454 Titanium sequencing platform.

### SNP discovery, validation and genotyping assay design

Sequencing reads were converted to FASTQ format and aligned directly to the *Phaseolus vulgaris* G19833 scaffold assembly v0.9 (originally available at phytozome.org) using GMAP [36] to produce SAM files. SNP discovery on the entire panel was undertaken using mpileup from Samtools version 0.1.18 (http://samtools.sourceforge.net/). After observing the degree of variation in the tepary lines, mpileup was run separately on the common bean and tepary bean lines to avoid algorithmic assumptions about population type and allele frequencies, as well as to simplify downstream filtering steps. Illumina's GoldenGate assays require that no other SNPs be present in the flanking sequence; so for the tepary assay design, alleles that were monomorphic in the two tepary lines were converted to the tepary base call in the flanking sequence prior to assay design.

No annotation information was available for the common bean scaffold assembly v0.9; alignments from all lines were processed through Cufflinks [37] to calculate the distance from each SNP to the exon boundaries. SNPs and associated flanking sequence were BLASTed against *Phaseolus vulgaris* v1.0 [28] (phytozome.org) once it was available to identify their position on the current genome assembly.

A set of 24 SNPs were chosen for validation (Additional file 2C) using in-house designed KASP assays (LGC Genomics, Hoddeston, UK). Allele-specific primer sets were designed and run using KASP reaction mix (version 3 chemistry, LGC Genomics) following the manufacturer’s instructions. PCR amplification was carried out in a StepOnePlus™ Real-Time PCR System (Applied Biosystems) and end-product fluorescence readings were analysed using StepOne Software v2.1 (Applied Biosystems). Genotyping results were compared to the expected SNP call based on the sequencing data.

**Fig. 1:**
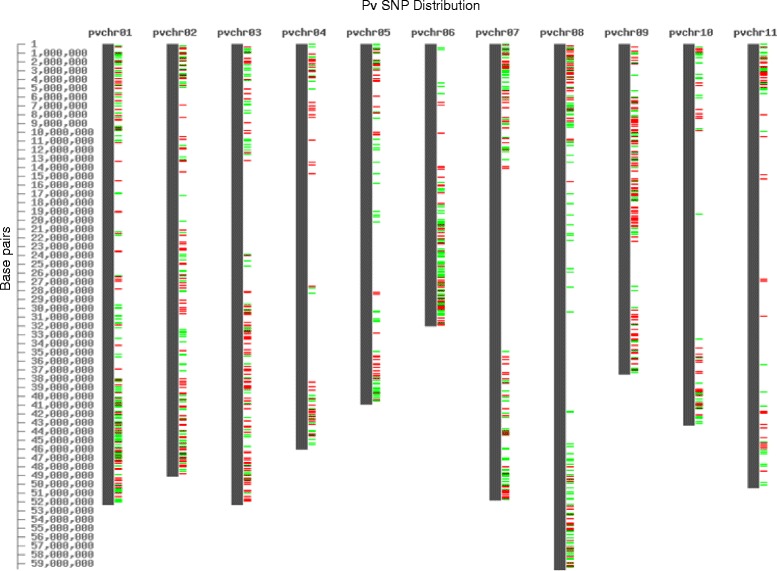
Distribution of SNPs selected for the common bean Pv768 (*green*) and tepary bean Pac768 (*red*). GoldenGate OPAs across the common bean pseudomolecules (v1.0, Phytozome.org)

SNPs with a minimum 60 bp flanking sequence were selected and sequences were submitted to Illumina’s (Illumina Inc., San Diego, CA) Array Design Tool (ADT) in order to obtain a designability rank score for each SNP ranging from 0 to 1. Due to the presence of large numbers of SNPs in flanking regions that were unique to tepary bean, two oligo pooled assays (OPAs) were designed: one for common bean (Pv768) and one for tepary bean (Pac768). SNPs were chosen from those with an ADT score greater than 0.6 as recommended by Illumina. SNPs were further reduced to 768 per species by selecting a subset that was reasonably evenly distributed across the genic regions of the *P. vulgaris* genome (Fig. 1). The Pv768 OPA had an emphasis on SNPs that would be polymorphic among Mesoamerican lines as this is the focus of breeding programs in Canada. These final sets were submitted to Illumina for design and synthesis of the OPAs. The Pv768 assay was used to genotype several different common bean populations and results have been reported separately [38]; here we report only on the results for the Mesoamerican and tepary genotypes used for SNP discovery.

### Phylogenetic analysis

The Pac768 OPA was used to genotype 156 tepary bean accessions, including W6 15578 and PI 430219, the source of the SNPs, and two *P. parvifolius* accessions (G40240; G40186), according to the standard Illumina GoldenGate assay protocol (http://www.illumina.com/documents/products/workflows/workflow_goldengate_assay.pdf). The products that were generated by this assay were read with an Illumina HiScan (Illumina Inc., San Diego, CA) and the resulting data were clustered for allele calling using GenomeStudio software version 2010.3 (Illumina Inc., San Diego, CA). The allele calls were manually inspected and corrected for misclassification of genotypes. Only markers having less than 10 % missing data were used for further analysis.

Phylogenetic analyses were performed using a parametric method based on the sequence alignment data. All SNP allele calls for the 158 genotypes were concatenated to give a specific sequence for each accession. Multiple sequence alignment was performed using ClustalW [39]; and the files were converted to MEGA format. A phylogenetic tree was constructed with MEGA6 [40] using the maximum-likelihood method and Kimura 2-parameter distance [41] for all substitutions. Gaps were treated as missing data using the “partial deletion” option, and the bootstrap consensus tree was inferred from 1000 replicates [42]. Branches corresponding to partitions reproduced in less than 50 % bootstrap replicates were collapsed and all positions with less than 95 % site coverage were eliminated. The phylogenetic tree was drawn and visualized using iTOL [43].

The genotypic data were analysed using the Bayesian clustering algorithm of STRUCTURE v2.3 [44] using the admixture model and correlated allele frequencies with a burn-in of 100,000 iterations, run length of 100,000, and K = 1 to 8. The optimal value of K was determined using the delta K procedure of Evanno et al. [45] using STRUCTURE HARVESTER v0.6.94 ([46], 2012; http://taylor0.biology.ucla.edu/structureHarvester/). The same analysis was subsequently performed on the cultivated and wild accessions separately.

### Genetic mapping of tepary bean and comparison with common bean

The Pac768 OPA was used to genotype 186F_2_ individuals of the BR-06 mapping population following the same protocol as for the diversity panel. The alleles were called as homozygous for one or other parent allele or heterozygous using GenomeStudio version 2010.3 (Illumina Inc., San Diego, CA), and were manually inspected and corrected for misclassification of genotypes. Unscorable markers and those missing the allele for one genotype (dominant) were discarded and only clearly polymorphic markers were mapped. Marker segregations were subjected to a Chi-square test to determine deviations from balanced segregation ratios for an F_2_ mapping population (1:2:1). Map construction was done using command line MST_MAP_ V4.3 [47, 48]. The genetic map thus generated was based on minimum spanning tree of a graph associated with the genotyping data, using a cut off p-value of 0.000001, COUNT objective function, and genetic distances were calculated using the Kosambi function [49]. Markers with zero recombination belong to the same genetic bin and those having the least missing data points were used to represent the bin. Linkage maps were drawn with the genetic-mapper Perl script (https://code.google.com/p/genetic-mapper). The map order matrix was visually inspected to confirm marker order. The order of the markers and orientation of the linkage group was further verified using MadMapper (http://cgpdb.ucdavis.edu/XLinkage/MadMapper/) with recombination value (haplotype distance) cut-off of 0.2 and a BIT score of 100. The visualization of the constructed map was done using CheckMatrix, which was obtained by running custom MadMapper Python scripts.

The SNP contigs mapped into linkage groups in BR-06 were mapped to *Phaseolus vulgaris* v1.0 (phytozome.org) and were processed through the NUCmer pipeline and the results were filtered for global alignment using length x identity weighted longest increasing subset [50]. Comparative dotplots were generated using MUMmerplot by parsing NUCmer output and visualized with Gnuplot and MS Excel.

## Results

### Large scale SNP discovery and validation

To capture a diverse subset of the genic nucleotide diversity in the common bean and tepary bean genomes, a set of six common bean and two tepary bean accessions were selected for targeted 3’-cDNA transcript profiling using 454 pyrosequencing technology. A total of 4,989,153 reads were generated across all eight genotypes (Table 1) with an average sequence length of 297 bases. In total, 3,684,523 (73.9 %) reads were mapped to the common bean v0.9 scaffolds. The average read depth sequenced from each genotype varied from 9 to 19. After filtering for read depth greater than 10 across all genotypes combined, 133,107 unique SNPs were identified in 1370 scaffolds (Additional file 2A). The final spreadsheet report indicates if the SNP is the same as the reference, the alternate allele, a third allele, or if there is no sequence data at that position. Once the common bean reference genome v1.0 was available, the flanking sequences for all SNPs were re-mapped and their locations on the pseudo-chromosomes established. There were 871 where the SNP position on the updated reference was unclear due to only a portion of the flanking sequence matching the v1.0 genome assembly.

Among common bean genotypes, most SNPs were associated with the Mesoamerican lines (Table 1). The tepary bean genotypes, W6 15578 and PI 430219, had 164,162 and 171,524 SNPs, respectively, relative to the common bean reference with 134,192 of these common to both tepary lines. After filtering there were 8471 SNPs between the two tepary bean genotypes (Additional file 2B). Across both tepary and common bean, 55 % of the SNPs were the result of transitions and 45 % were transversions.

To validate the high confidence SNPs identified in this study, 24 KASP single-SNP assays were designed and amplified on the eight sequenced genotypes. Allele calls for 22 of them matched the predicted allele based on the 454-derived sequences. PvSNP22p781281 had a mismatch in CDC WM-2 and PvSNP238p102141 had a mismatch in W6 15578 (Additional file 2C). Results for PvSNP390p263061 did not match the 454 SNP call for SMARC1-PN1 or W6 15578, but in both cases the number of sequence reads fell below our confidence threshold of three reads for the individual so the KASP result may in fact be the correct allele call. Most of the failed assays were within the tepary bean line W6 15578; eight did not produce a product. Of these, five also did not have a 454 read and the other three were below the threshold for calling an allele from the 454 sequence. Two loci that did not have 454 allele calls did amplify with the KASP assay. The other tepary bean line, PI 430219, had only three KASP assays fail; none of which had a confident 454 call and all three of which also failed in W6 15578.

**Fig. 2:**
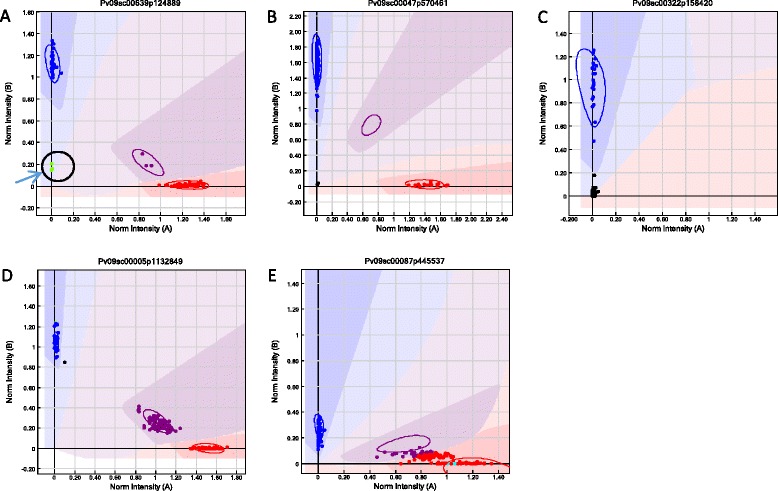
Examples of SNP locus results as visualized using GenomeStudio version 2010.3 (Illumina Inc.). **a** Three tepary bean genotypes (*circled green dots*) falling outside the cluster of common bean genotypes for PvSNP639p124889 from the Pv768 OPA; **b** polymorphic marker PvSNP47p570461 on 158 tepary bean accessions genotyped with the Pac768 OPA; **c** dominant marker PvSNP322p158420 on 158 tepary bean accessions genotyped with the Pac768 OPA; **d** polymorphic marker PvSNP5p1132849 on BR-06 F2 individuals genotyped with the Pac768 OPA showing 1:2:1 segregation pattern; **e** questionable and difficult to score marker PvSNP87p445537 on BR-06 F2 individuals genotyped with the Pac768 OPA

The loci represented on the two separate GoldenGate OPAs cover the genic regions of common bean (Fig. 1) [28]. The allele calls for CDC WM-2, Expresso, Higuera-E, SMARC1N-PN1, W6 15578 and PI 430219 based on the Pv768 OPA are presented in Additional file 2D. Allele calls matched the expected genotype based on the 454 sequencing data for 86–93 % of the loci for the common bean lines. When missing and questionable 454 calls were removed, over 99 % of the SNP allele calls matched the expected allele from the 454 data. The tepary bean lines were much less successful on this OPA, with fewer than 60 % of the loci returning a matched allele call. Most of the other loci failed to produce a result with DNA from these lines. The tepary results sometimes clustered with lower levels of intensity than the common beans (e.g. Fig. 2a) suggesting problems with primer annealing and further confirmed the need for a separate assay for tepary bean. When the two tepary beans were genotyped with the Pac768 OPA, greater than 90 % of the loci returned the expected allele call and fewer than 4 % of the loci failed (Additional file 2E).

### Phylogenetic analysis of tepary bean accessions

Genotyping of the tepary bean germplasm collection with the Pac768 OPA resulted in 563 of the SNPs falling into two clear main clusters representing the two homozygous genotypes (e.g. Fig. 2b). There were 94 SNPs that had a small additional cluster in the middle of the graph corresponding to heterozygous/heterogeneous genotypes. Five dominant markers were identified, where one allele clustered and other failed (e.g. Fig. 2c); all were included in the diversity analysis with the other polymorphic markers. An additional 66 (8.6 %) markers were monomorphic, 20 (2.6 %) were too difficult to score and 20 (2.6 %) markers failed to amplify. From the total 662 clean polymorphic markers, only the 645 markers having less than 10 % missing data were used for phylogenetic analysis.

**Fig. 3:**
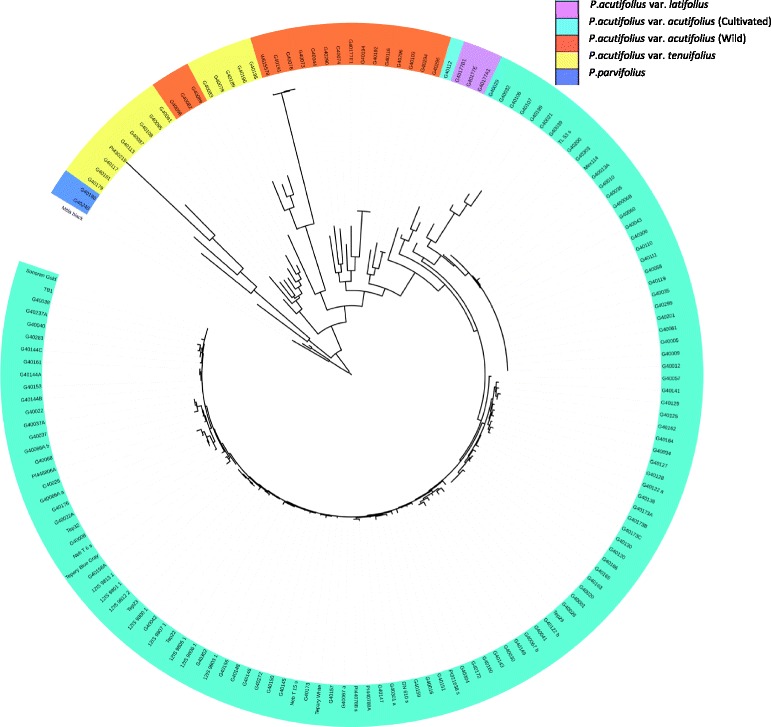
Phylogenetic relationships among tepary bean (*P.acutifolius* var. *actutifolius* (cultivars), *P.acutifolius* var. *acutifolius* (wild), *P.acutifolius* var. *tenuifolius, P.acutifolius* var. *latifolius*) and *P. parvifolius* accessions based on genotypes from 645 SNP markers assayed using the Pac768 OPA. Analysis was based on sequences generated by concatenating SNPs, aligned using ClustalW [39] and the tree was constructed using the maximum likelihood method with 1000 bootstrap in MEGA6 [40]

Phylogenetic relationship analysis resulted in a bifurcated tree with the cultivated tepary beans separating completely from the wild accessions (Fig. 3). The 116 cultivated genotypes formed a tightly linked cluster which subdivided into two major sub-clusters. These sub-clusters were generally low in diversity and separated based on geographic origin of the genotypes: Central America or USA/Mexico with a few interspersed from African regions (Additional file 1). Within the wilds, subgroups were identified that more or less corresponded to that expected based on taxonomic classification with a few exceptions. There were three clusters of var. *tenuifolius*, two of which also contained var. *acutifolius* accessions. One set of five var. *tenuifolius* lines were much more diverse and separated out completely from the others. The var. *latifolius* lines fell between the cultivated and the wilds. *P. parvifolius* formed a distinctly separate cluster. One line, Mitla Black, separated from all the other lines that were genotyped.

**Fig. 4:**
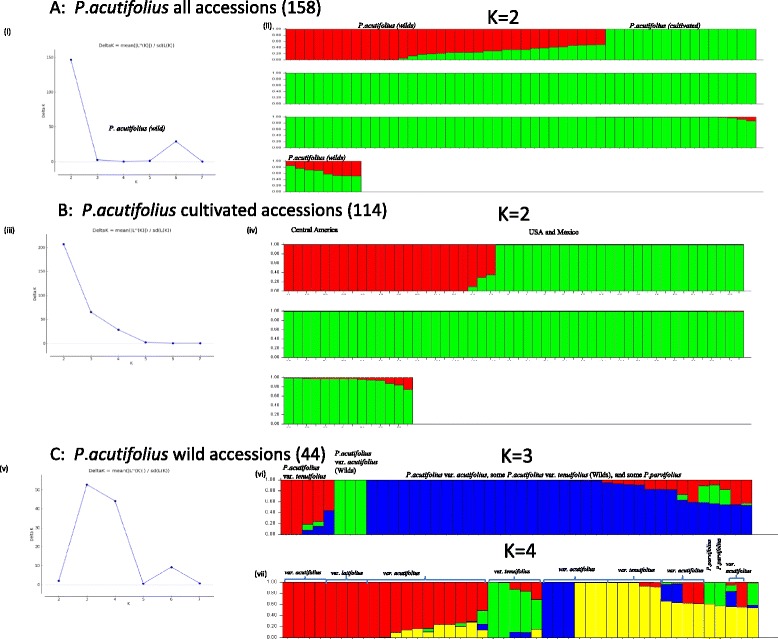
Population structure analysis of *P. acutifolius* and *P. parvifolius* accessions showing clustering of individuals into populations on the basis of multi-locus genotyping using STRUCTURE v2.3. **a** all accessions; **b**. cultivated *P. acutifolius* accessions only; **C**. wild *P. acutifolius* accessions only. (i,iii,v): optimal number of populations (K) for each set, calculated and displayed graphically using STRUCTURE Harvester [46]. (ii,iv,vi,vii): STRUCTURE output for each set of accessions. Each color represents one population and each accession is represented by a vertical bar. The length of each colored segment in a vertical bar represents the proportion of alleles contributed by each of the populations

STRUCTURE analysis on the wild and cultivated accessions distinguished two sub-populations: cultivated and wild (Fig. 4a) which corresponded to the major groups in the phylogenetic tree. To further evaluate the genetic structure of the two sub-populations, the groups were re-analysed separately. Analysis of the cultivated tepary beans supported the two sub-population model observed in the phylogenetic tree with very little admixture (Fig. 4b). In case of the wild accessions, the optimal K appeared to be three or four (Fig. 4c). At K = 4, the major phylogenetic lineages were resolved according to those expected based on the phylogenetic tree. There was a group of three wild var. *acutifolius* accessions (Fig. 4c, blue), including W6 15578, that separate out from the rest of the wild var. *acutifolius* (Fig. 4c, mostly red and yellow). The var. *latifolius* accessions form a group with several var. *acutifolius* accessions (Fig. 4c, red). Results indicate that var. *tenuifolius* is separated into two groups one of which overlaps with var. *acutifolius* (Fig. 4c, yellow). A similar split was seen in the phylogenetic tree (Fig. 3). *P. parvifolius* shares alleles with both var. *tenuifolius* groups (Fig. 4c, green and yellow).

### Genetic mapping of tepary bean and comparison with common bean

Genotyping results from the BR-06 F_2_ mapping population revealed three clusters: one for each parent allele and one for heterozygotes (e.g. Fig. 2d), for 678 (88.3 %) markers. Five of these were significantly distorted (*P* < 0.01) from the expected 1:2:1 segregation ratio and in preliminary mapping were only loosely linked to two linkage groups. These were discarded leaving 673 loci for final mapping. There were 92 SNPs that were not useful for mapping: 56 (7.3 %) failed to amplify, 22 (2.9 %) were monomorphic, five appeared to show the presence of a gene duplication and could not be scored (e.g. Fig. 2e) and seven had a dominant segregation pattern which cannot be easily scored in an F_2_ population.

**Fig. 5:**
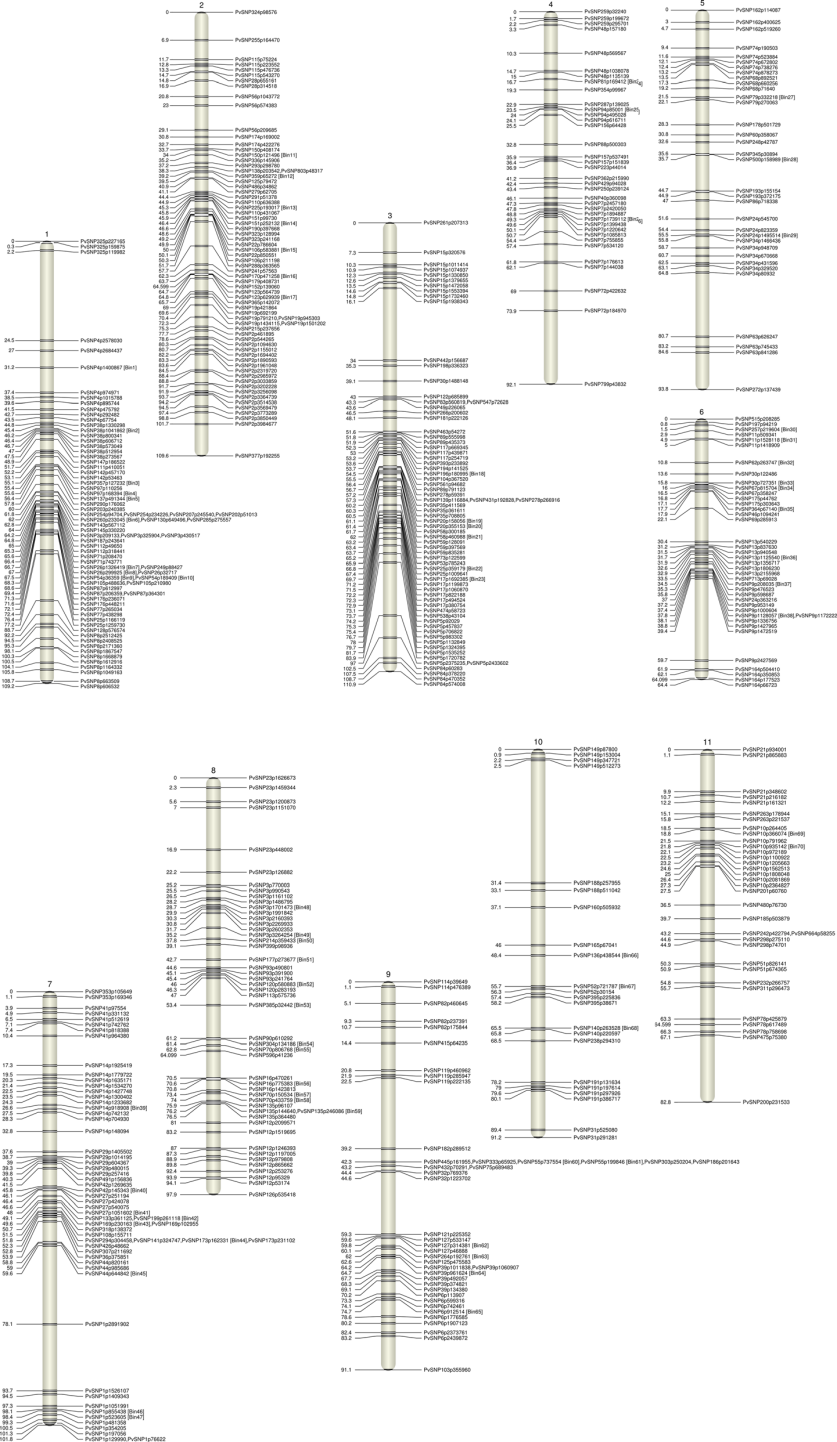
Gene based linkage map of *P. acutifolius* based on the F_2_ mapping population BR-06 derived from the cross W6 15578 x PI 430219. Genetic distance between markers (cM) are indicated on the left of the linkage group and the locus names are on the right. Bins having >1 markers are indicated in brackets beside the locus that was mapped

Markers were binned based on identical scoring patterns, resulting in 70 bins with greater than one member and 459 singleton loci. These 529 loci grouped into 11 linkage groups (Fig. 5; Table 2; Additional file 3). The linkage groups were numbered based on shared synteny with the pseudochromosomes of the *P. vulgaris* 1.0 genome assembly. Linkage groups varied from 64.4 cM (LG6) to 110.9 cM (LG3) in length spanning 1044.9 cM in total. There were between 22 and 74 markers per linkage group resulting in an average marker density of 1.97 per cM across the genome. Linkage group 9 had a large number of markers that had zero recombination. There were only two gaps over 20 cM: on LG 1 and LG10. Linkage order was confirmed using Checkmatrix in MadMapper (Additional file 4) and the linkages spanning these two gaps are most likely correct.

**Table 2:** Summary of distribution of SNP markers and bins in tepary bean linkage groups

Linkage Groups (LG)	Markers Represented in Map	Total no of recombination Bins per LG	No of additional markers in bins	Total mapped markers	Total distance covered in cM
LG1	74	10	15	89	109.2
LG2	70	7	9	79	109.6
LG3	71	6	12	83	110.9
LG4	36	3	3	39	92.1
LG5	34	3	4	38	93.8
LG6	40	9	12	52	64.4
LG7	58	9	9	67	101.8
LG8	49	12	16	65	97.9
LG9	41	6	57	98	91.1
LG10	22	3	4	26	91.2
LG11	34	2	3	37	82.8
Total	529	70	144	673	1044.9

The tepary bean linkage groups aligned to individual common bean chromosomes with very few single marker exceptions (Fig. 5; Additional file 3). There were no inter-chromosomal translocations evident. The only differences observed between the two genomes were restricted to intra-chromosomal inversions and translocations with respect to common bean, mainly in tepary bean linkage groups 2, 3 and 9.

## Discussion

Tepary bean is an important crop both as a source of interesting variability for common bean via interspecies hybridization but also as another domesticated bean crop for areas of the world where environmental stressors make common bean a risky crop choice. To facilitate breeding with this species, there is a need to develop modern genomic resources. The current molecular marker resources for tepary bean are restricted to a few SSRs and SNPs from common bean and AFLPs, neither of which is ideal for genetic studies nor for breeding purposes. When we started this work there were very few SNP markers for common bean and no sequenced genome. There are now many SNPs identified in common bean [22–24, 51–53] and a fully sequenced genome [28].

### SNP mining and genotyping

The Roche 454 FLX technology delivered large amounts of long read data and provided an effective means to generate the sequence resources required to assemble a panel of SNPs for genotyping experiments. The panel of genotypes was chosen to represent a range of common bean types (both Andean and Mesoamerican) as well as two tepary beans. Initially, CDC WM-2 was selected as reference genotype for which additional sequencing was carried out with the idea to develop a reference *de novo* assembly to which the other genotypes would be compared for SNP identification (Table 1). With the availability of a preliminary assembly of the G19833 common bean genome (v0.9), the sequence reads were instead aligned to this as the reference genome. On average, 74 % of the 3’ reads uniquely mapped to the v0.9 reference assembly. This high level is due to the sequences being derived from expressed genes and the diploid nature of the bean genome. Version 0.9 was a set of 10,132 scaffolds that had yet to be anchored to pseudomolecules or tied to known chromosomes and some scaffolds were re-arranged in the final v1.0 assembly. Sequences flanking the SNPs from v0.9 were used to identify the location of the SNPs in v1.0 (Additional file 2A) and these rearrangements are likely responsible for the inability to find corresponding locations for 2826 SNPs in v1.0.

The Mesoamerican genotypes had more SNPs than the two Andean genotypes, which is understandable given the reference genome is an Andean type and the long history of divergent selection between these two gene pools of bean. The majority of the SNPs were found in the two tepary bean genotypes which is not unexpected for a distant relative, but the number was very high.

SNP validation was successful for the common bean genotypes but much less so for the tepary beans where several assays failed to work. The most likely reason is the presence of SNPs in the primer binding regions that prevented complete hybridization and amplification. Many that failed also did not have a 454 allele call in tepary bean suggesting the sequences from corresponding regions in tepary did not align sufficiently well to the reference genome to be included in the dataset. This exposed the need to develop separate genotyping assays for the two species. This was confirmed by the success of genotyping common bean (>85 % loci reporting) vs tepary bean (<60 % loci reporting) lines with the Pv768 OPA. Goretti et al. [22] successfully amplified tepary bean DNA with several KASP-based SNP assays but this could be due to them choosing conserved orthologous genes for their assays. By their nature, these genes should have higher levels of cross-species homology. In contrast, the tepary bean lines were successful for 96 % of the loci with the Pac768 OPA. These results are in the range of other legume crops for this type of assay: lentil (84 %; [35]), pea (96 %; [54]), chickpea (99 %, [55]), and soybean (80 %, [56]).

### Genetic diversity and population structure analysis

Understanding the genomic relationships amongst diverse germplasm is essential for efficient use of genetic diversity in a crop improvement program. The type of molecular markers used in diversity analysis plays a critical role in predicting the relationships among different accessions. While several diversity studies have been done in tepary bean, they have relied on phenotypic [57], protein [3, 58], AFLP markers [29] or common bean-derived SSR markers [26] and have limited genome coverage. SNPs have become the first choice for diversity studies and association mapping due to their high abundance across the genome and their ability to sample diversity [59, 60]. The Pac768 OPA described here assays diversity in 768 genes across the genome (Fig. 1).

Both cultivated and wild accessions of *P. acutifolius* and its wild sister species *P. parvifolius* were genotyped using the Pac768 OPA and the majority of the SNP loci returned clear bi-allelic profiles, indicative of homogeneous populations. In contrast to previous studies, the SNPs surveyed here are gene-based, so have a higher probability of representing functional variation. The cultivated teparies were less diverse than the wilds, something already noted by others based on phaseolin pattern [58], isozymes [3], and molecular markers [26, 29]. Genetic diversity assessments based on both STRUCTURE and phylogenetic inference confirmed that the domesticated tepary beans were genetically more closely related to one another than to their wild relatives and clearly separated into two groups based on their eco-geographical origin with little admixture (Figs. 3 & 4). Tepary bean is though to have undergone multiple domestications [61] and these results suggest the possibility of one in each of Central America and Mexico/USA. That there is one distinct separation between the two domesticated groups and the wild accessions suggests, however, that it is more likely that there was an early domestication event followed by separation based on region. The Central American accessions were more diverse than the Mexican/US ones but this was due to only a few of the genotypes; otherwise a large number were genetically very similar (Fig. 3).

Tepary bean as a crop has traditional origins in the arid southwest USA and northern Mexico but has expanded its range due to its success in stressful environments. The accessions that were collected from Africa clearly trace back to this area, likely as a result of testing germplasm for performance in similar growing regions in order to introduce a nutritious, stress-tolerant, warm-season legume to a new region. The accessions from Zimbabwe, G40302 and G40301, showed most similarity with the Mexican cultivars G40156 and G40151 (Sonora), respectively. A Zambian accession, G40122, and G40041 from South Africa, were most similar to the Mexican cultivar G40138 (Sinaloa). The accession from Morocco, G40008, was very similar to the USA cultivars, G40068 and PI 448806a (Arizona). In a similar fashion, three of the four cultivars that were obtained in Saskatchewan, Canada, are most similar to various tepary beans from Mexico and the USA. The fourth SK cultivar was not at all related to the domesticated accessions but formed an out-group with the *P. parvifolius* accessions (Fig. 3). In the field it is clearly domesticated, with large seeds, no dehiscence, and has leaves that are more reminiscent of var. *acutifolius*, definitely not *P. parvifolius*, and, therefore, is more likely something more distantly related, perhaps a common bean. In fact, there is an accession in GRIN (PI 550234, http://www.ars-grin.gov/cgi-bin/npgs/acc/display.pl?1445170) called ‘Mitla Black’ that is classified as *P. vulgaris*. Unlike the other tepary beans that were genotyped using the Pv768 OPA, the Saskatchewan version of ‘Mitla Black’ always clustered within the common bean allele cluster and never with the tepary beans when they fell outside the main allele cluster (Fig. 2a) further suggesting it is more likely a common bean than a tepary bean. Blair et al. [26], had reported G40272, a white seeded tepary accession from Sonora, as possibly being a misclassified wild genotype, but in both Muñoz et al. [29] and our study, it clearly falls within the cultivated genepool as designated in genebank databases.

The var. *latifolius* lines that were genotyped were a set of four sublines from a weedy accession (G40177) that was a mix of different types. As suggested by Pratt and Nabhan [61], var. *latifolius* is a “*nomum confusum*” for var. *acutifolius*. One of the four (G40177E1) was genotypically more similar to the wild var. *acutifolius* accessions and three (G40177A1, G40177B1 and G40177E) formed a distinct cluster within the cultivated var. *acutifolius* group from Central America, suggesting it is a feral var. *acutifolius*.

The SNPs on the Pac768 OPA were identified between PI 430219 a var. *tenuifolius* accession from New Mexico, USA, and W6 15578, a wild var. *acutifolius* accession from Mexico, and these two form extremes of the wild accessions (Fig. 3). Closely related to W6 15578, were G40191 and G40076, both from New Mexico and all three formed a unique var. *acutifolius* population within STRUCTURE (Fig. 4c - blue). The majority of the other wild var. *acutifolius* accessions showed evidence of admixture with alleles in common with var. *tenuifolius* (Fig. 4c). There were three accessions labeled var. *acutifolius* that clearly fell with several var. *tenuifolius* accessions: G40096, G40082 and G40089. All are morphologically more similar to var. *acutifolius* with non-lobed lateral leaflets in contrast to var. *tenuifolius*, which typically have lobed lateral leaflets [12] so it is doubtful they are misclassified. All were from Durango, Mexico as were the var. *tenuifolius* accessions they grouped with. Blair et al. [26] genotyped two of these accessions and they were also found to be distinct from most other var. *acutifolius* but were closely related to var. *latifolius* which is not evident from our analysis. As with the findings of Blair et al. [26], there were two distinct groups of var. *tenuifolius*, the one from Durango and a second, more diverse group from Arizona, USA.

*Phaseolus acutifolius* and *P. parvifolius* are considered to be sister species and *P. parvifolius* and *P. acutifolius* var. *tenuifolius* are often mistaken for one another [12]. Muñoz et al. [29] used AFLP markers to demonstrate that *P. parvifolius* is distinct from *P. acutifolius* in relation to other *Phaseolus* spp.. STRUCTURE analysis using the SNP data suggested that the two *P. parvifolius* accessions (G40240 and G40186) had alleles in common with the two var. *tenuifolius* groups (Fig. 4c), suggesting that these are ancestral alleles that were selected in one or other of the two different *tenuifolius* groups.

### First gene-based linkage map of tepary bean and comparative mapping with common bean

**Fig. 6:**
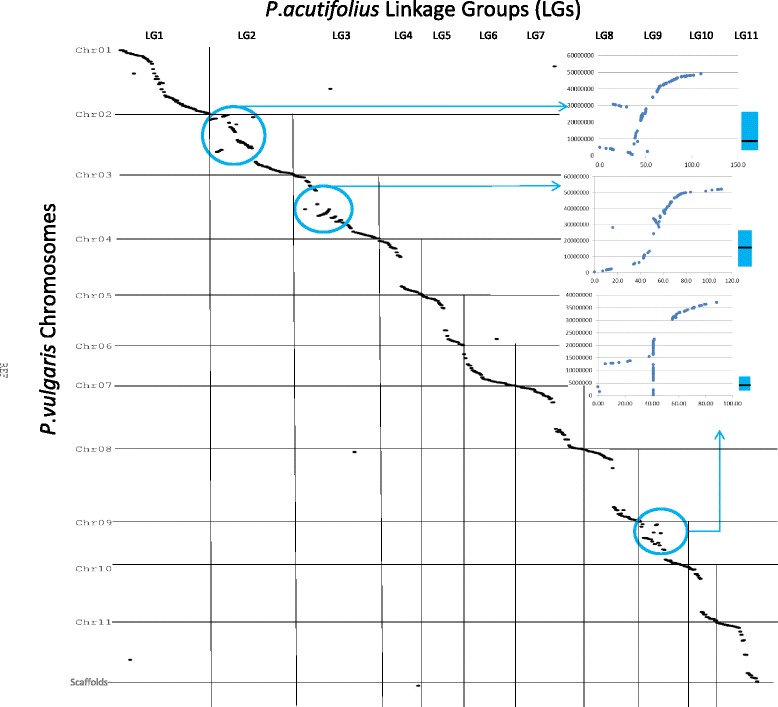
Dot plot representing correspondences between *Phaseolus acutifolius* linkage groups LG1 to LG11 (*top*) and *P. vulgaris* chromosomes Pv01 to Pv11 (*left side*) based on alignment of tepary bean sequences to *P. vulgaris* v1.0 and visualisation using NUCmer and MUMmer plot software [50]. Major translocations and re-arrangements are circled in blue. Plots of *P. acutifolius* genetic distance vs *P. vulgaris* physical distance for LG2, LG3 and LG9 are highlighted on right. Blue bars within these plots represent the *P. vulgaris* pericentromeric regions with the centromere marked by a black band based on Schmutz et al. [28]

Construction of a genetic map is a valuable first step to better understanding genome organization in species without a sequenced genome. The only published genetic map involving tepary bean thus far has been an AFLP map of an interspecific population derived from a cross between common bean and tepary bean [62]. The BR-06 population is from an intraspecific cross between the tepary genotypes used to generate the SNP panel: W6 15578 and PI 430219. The resulting map consists of 673 loci including bin loci that mapped into 11 linkage groups, likely representing the 11 chromosomes of this species. The SNPs assayed are all gene-based and the locations of homologues in the common bean genome are known, making them useful for future gene-based marker discovery. The large cluster of loci on LG9 is likely the result of an inversion that is present in one of the tepary bean parents relative to the other. Inversions and translocations will inhibit recombination and prevent genetic mapping of loci that fall within that region of the genome. This is the case in *Medicago truncatula* where crosses between several different accessions and the sequenced line, A17, result in a similar inability to map a region of chromosomes 4 and 8 due to a translocation within A17 [63]. This cluster of markers corresponds to a region on the common bean chromosome 9 covering approximately 23 Mbp and some appear to be part of a translocation event relative to common bean (Fig. 6). That there is an inversion in this region is not surprising given that there is already evidence of breakpoints in this region.

When BR-06 was genotyped using the Pac768 OPA, 22 loci were monomorphic. This could be the result of designing primers based on the common bean sequence with inferred bases from the tepary bean sequences, which could lead to amplification of a paralogous gene that does not have this SNP. It could also be due heterogeneity in the original accession at the locus and the use of different plants for the cross than for the SNP identification. Only five markers (0.7 %) were significantly distorted (*p* < 0.01) suggesting that neither parent was favoured during recombination in any region of the genome surveyed. This is even lower than the 10 % observed in the tepary bean F_2_ populations reported by Garvin and Weeden [3].

High levels of conserved macro-synteny have been observed among legume genera [35, 64–67] and within genera, the extent of conserved synteny is further extended (e.g. *Glycine max* and *G. soja* [68]; *Lens culinaris* and *L. ervoides* [69]; *Medicago truncatula* and *M. sativa* [70]. The base chromosome number in the genus *Phaseolus* is x = 11 [71, 72] and most species are diploid with 2n = 22 chromosomes. Tepary bean is estimated to have a slightly larger genome (647 Mbp) than that of Lima bean (*P. lunatus*, 622 Mbp) and common bean (637 Mbp) [73]. Thus far, comparative mapping among *Phaseolus* spp. has been restricted to cytogenetic analyses based on C-banding patterns [74] and hybridization of common bean BACs and repetitive DNA fragments to chromosomes of other species [13, 14, 75] with a focus on *P. lunatus* and the wild *P. microcarpus*.

The majority of the SNP markers used to develop the tepary bean map have physical locations in the common bean genome. This allowed for the direct comparison of these two species at the gene level and demonstrated high levels of conserved synteny between these two species (Fig. 5). Low levels of recombination due to the presence of the highly repetitive DNA were observed around pericentromeric regions in *P. vulgaris* [76, 77] and *P. lunatus* [13]. The gaps on linkage groups 4, 5, 7, 8, 10 and 11 observed in the tepary genetic map relative to the common bean physical map (Fig. 6) can be explained by the presence of these gene-deficient, pericentromeric heterochromatic regions in each of the common bean chromosomes [28].

The rearrangements of tepary bean linkage group 2 relative to common bean chromosome 2 consist of an inversion and a translocation from the middle of the inversion (Fig. 6 inset A). The translocated segment is also inverted suggesting the translocation happened after a large inversion. This inversion terminates proximal to the pericentromeric region of common bean. The main difference between linkage group 3 and common bean chromosome 3 is an inversion in the middle of the chromosome, distal to the pericentromeric region (Fig. 6 inset B). The rearrangement on linkage group 9 is more complicated to interpret due to the large region that cannot be mapped due to zero recombination but it involves a large translocation around the pericentromeric region in common bean (Fig. 6 inset C).

Cytogenetic comparisons between *P. vulgaris* and *P. lunatus* chromosomes based on hybridization of common bean BACs revealed three inversions, on chromosomes 2, 9 and 10 [13]. Cytogenetic observations of the same BACs hybridized to *P. microcarpus*, a distant wild relative, indicated the presence of four breaks in collinearity, likely due to inversions [14]. Three of these inversions involved chromosomes 2, 3 and 9 suggesting these could have occurred in the common bean lineage after it split from the common ancestor with the closer relative, *P. acutifolius*. Verification of this would require mapping gene-based markers in *P. lunatus* and *P. microcarpus*, however. Cytogenetic analyses using these common bean BACs on cowpea (*Vigna unguiculata*; [78]) suggest that beyond the *Phaseolus* spp. level, inter-chromosomal translocations begin to define differences along with inversions. The much larger number of markers that were used in the tepary bean – common bean comparison illustrated here builds on these cytogenetic observations and gives confidence to the findings that the major differences among these species are related to very few major intra-chromosomal rearrangements.

Tepary bean is in the tertiary gene pool of common bean and interspecies crosses between the two require embryo rescue for several generations to be successful. Despite this, there are examples of tepary bean being used as a source of novel allelic variation for common bean breeding; most notably for tolerance to common bacterial blight [7–9]. The high level of collinearity combined with large amounts of variation between the two species demonstrates that mining either species to improve the other should be possible across a large portion of the genomes.

Having gene-based markers with defined positions in better-studied relatives leads to the ability to predict where genes of interest may lie or match QTL with those found in other species that are better characterized. Knowledge of the genomic relationships between homologous chromosomes and the availability of the common bean genome sequence provides an important genomic resource for the less well studied tepary bean. One thing to bear in mind when using the common bean genome, however, is the existence of a large number of SNPs relative to the common bean sequences, which must be taken into consideration when designing primers based on common bean sequences for use in tepary bean.

## Conclusions

Tepary bean is a promising crop for semi-arid environments and its tolerance to various stresses, both biotic and abiotic makes it more tolerant to variable climate and of interest as a source of genetic variability for common bean improvement. Limited genomic resources are the main hurdles in the improvement of tepary bean. Therefore, there is a need for the development of resources to carry out genome-wide profiling and trait-specific marker-assisted selection.

This study provides a large selection of transcript-based SNP markers for use in various applications for both tepary bean and common bean. They will be useful for mapping in both crops and offer the ability to track introgression of segments of one into the other following interspecies hybridization. The strong collinearity observed between the two species suggests it should be possible to introgress some of the beneficial alleles from one into the other with the possible exception of those found in the few rearranged segments on chromosomes 2, 3 and 9. Confirmation of this will be possible through the use of the SNP markers to genotype individuals from intraspecific crosses. Resources for common bean genetic research are much greater than those for tepary bean but it should be possible to leverage information from this fully sequenced species for molecular marker-based breeding and gene discovery in tepary bean.

### Data availability

All sequencing and SNP data related to this project are available through the project page on our KnowPulse web portal (http://knowpulse2.usask.ca/portal/project/Phaseolus-vulgaris-454-Sequencing-and-SNP-Discovery-Project). In addition, all sequence data produced from the 454-FLX have been deposited in the NCBI, Sequence Read Archive [GenBank Bioproject ID: PRJNA285249; SRX1047109-SRX1047116].

### Ethical standard

The authors declare that the experiments of this study comply with the current laws. We confirm to have the authority to publish this work and that the manuscript has not been published before and is not under consideration for publication elsewhere.

## Additional files

Additional file 1: List of *P. acutifolius* accessions selected for phylogenetic analysis. (XLSX 19 kb) Additional file 2: A) List of SNPs identified in 6 *P. vulgaris* and 2 *P. acutifolius* genotypes along with the corresponding homologues position in Pv1.0 assembly, B) SNPs exclusive to teparies, C) results of SNPs selected for KASP assay, D) Pv768 GoldenGate assay results, E) Pac768 GoldenGate assay results, and F) summary of assay results. (XLSX 20503 kb) Additional file 3: A) BR-06 map with binned marker loci and corresponding *P. vulgaris* pseudochromosome locations and B) SNP genotypes for 158 accessions using Pac768 GoldenGate assay. (XLSX 497 kb) Additional file 4: Two-dimensional heat plot of pair-wise genetic distances among the loci in the 11 tepary bean linkage groups. Each linkage group is plotted in the linear order of the loci against itself and the degree of linkage between each locus indicated by a color. Red indicates tight linkage; green, unlinked. The first column at the right lists the locus name. The second column indicates the genetic distance. (DOCX 455 kb)

## Abbreviations

ADT: array design tool (Illumina Inc)
CBB: common bacterial blight
CDC: Crop Development Centre
CIAT: International Centre for Tropical Agriculture
MAS: marker-assisted selection
NGS: next-generation sequencing
OPA: oligo pooled assay
SK: Saskatchewan
SNP: single nucleotide polymorphism
