# Supplementary material for: Gene-based SNP discovery in tepary bean (Phaseolus acutifolius) and common bean (P. vulgaris) for diversity analysis and comparative mapping
Source: BMC Genomics. 2016 Mar 15;17:239. doi: 10.1186/s12864-016-2499-3 (PMC4793507; doi:10.1186/s12864-016-2499-3)

**Additional file 4:** Two-dimensional heat plot of pair-wise genetic distances among the loci in the 11 tepary bean linkage groups. Each linkage group is plotted in the linear order of the loci against itself and the degree of linkage between each locus indicated by a color. Red indicates tight linkage; green, unlinked. The first column at the right lists the locus name. The second column indicates the genetic distance.


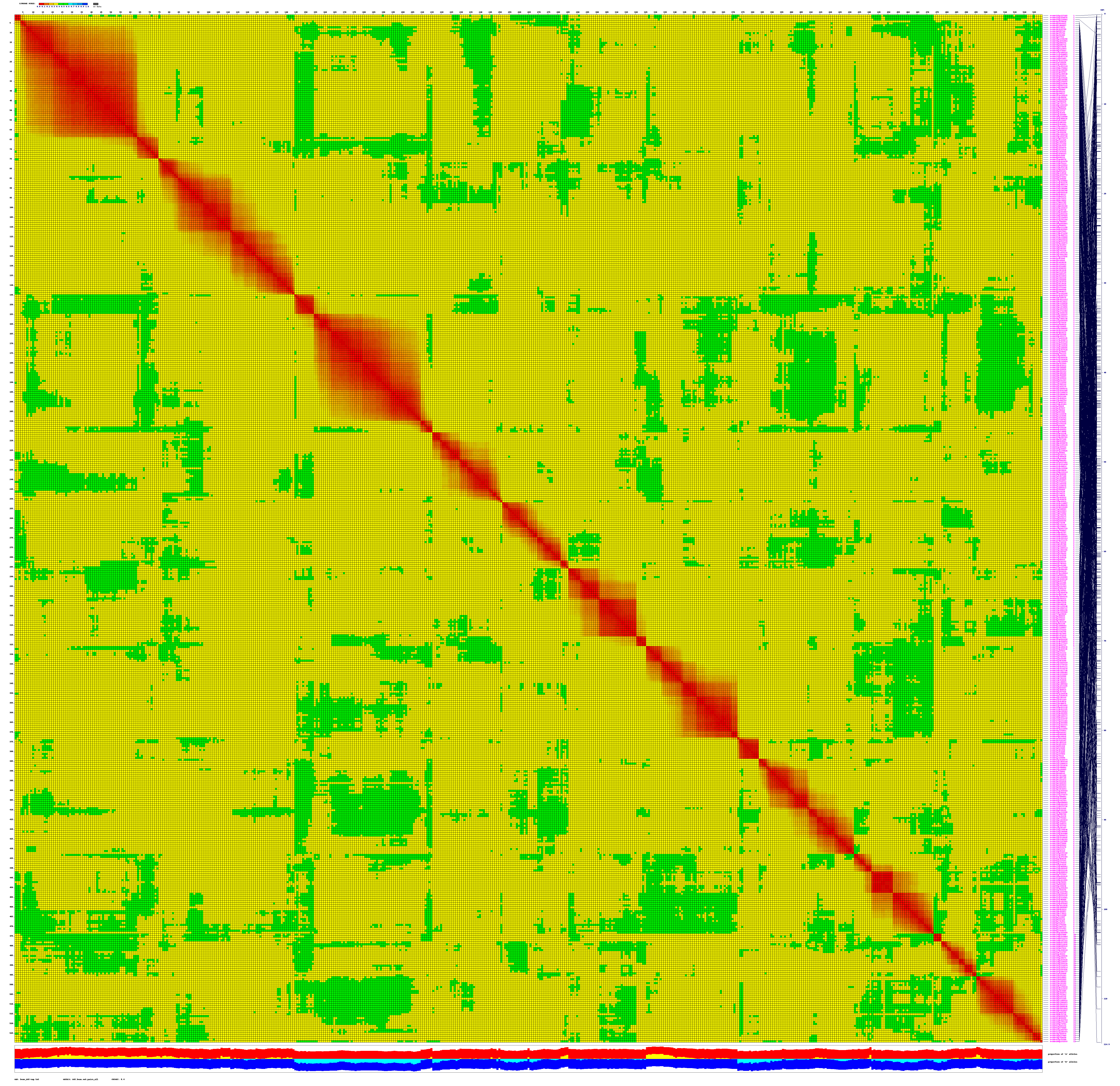

Supplement: Additional file 4: — Two-dimensional heat plot of pair-wise genetic distances among the loci in the 11 tepary bean linkage groups. Each linkage group is plotted in the linear order of the loci against itself and the degree of linkage between each locus indicated by a color. Red indicates tight linkage; green, unlinked. The first column at the right lists the locus name. The second column indicates the genetic distance. (DOCX 455 kb) [file 12864_2016_2499_MOESM4_ESM.docx]
